# London Baby Week

**Published:** 1918-07-13

**Authors:** 


### LONDON BABY WEEK.

### Some Lessons of the Conference.

By A SOCIAL WORKER.

"If," said Sir Owen Seaman, "you want to secure yourself by protective colouring against the assaults of old age, work for the children." The counsel is not, after all, so purely self-regarding as it sounds, for as we come in the course of such a conference to dissect—or, rather, to build up one by one the items of which our task for the future is made up—we crave for time to carry it out. "Show Thy servants Thy work and their children Thy glory."

Lady Barrett hoped the figures of our infant death-rate and damage-rate were *still* alarming. It may be true that constant repetition makes us callous, or that we may from weariness be glad to say that things have been very much improved, and turn contentedly back to some war work of the present. If so, the discussion on statistics—one which would have rejoiced the heart of Florence Nightingale—was calculated to kill self-satisfaction. The work of Dr. Ballantyne, of Edinburgh, from which he in youth was discouraged by his elders and teachers, as was Pasteur in his inquiries, shows that accurate knowledge of figures needs to be gained and laid as the first foundations of "the new activity of baby-saving." Mortality tables do not count the still-birth and miscarriage, and "till the total number of babies entering for the race of life" is known it is not possible to devise adequate remedies. We do not know the sterility rate. We have learnt that the syphilitic are fertile, while gonorrhœa produces the opposite effect, but what percentage of loss to attribute to the former by deaths from premature or post-natal weakness is not clear, nor is the incidence of certain poisonous trades such as work in lead and mercury upon fathers, or certain industrial occupations among mothers, as productive of the same results. We dimly perceive what a sound temperance campaign may do when we hear such results as Lieut.-Col. Adami, of Montreal, stated from Dr. Stockard's Cornell experiments with alcoholised guinea-pigs. Not only did two alcoholised grandfathers reduce in the third generation a normal progeny of 100 to five weakly specimens, but in some cases eyes were not developed.

But if our knowledge is incomplete, it is sufficient to indicate certain very definite immediate duties. The first is perhaps pre-natal care for the bodies and training for the minds of working-class mothers. All testimony, notably that of their own letters, goes to show that once she understands, the mother can and will do a great deal for herself and the child. Details of its clothing, feeding, and daily management can be learnt in the waiting-time. Some of these, as school-lessons in mothercraft encouragingly show, can be learnt by little girls, and the lesson is most popular. We should like to add that some items are not beyond the grasp of boys. One of the most interesting studies in street life is the occasional devoted care of a little child by a bigger brother. A quaintly suggestive hint of teaching possibilities was given by Dr. Knox, U.S.A., in his account of American Red Cross work in France. Punch-and-Judy shows have been so adapted as to teach a lesson more up to date than their old story of infant ill-usage and its consequences.

Next comes the necessity that birth should take place in fit surroundings. For the many who are at present homeless, such as the young wives of soldiers who have not set up a home, and the unmarried mother, and for the case known to be abnormal, an institution may be best. But the mother who can have what Lady Barrett described as the minimum, a free room and proper provision of linen and of water, is happier at home with the family purse under her pillow and the children within call. Only she needs the good midwife, and it is not satisfactory to find that out of 40,000 enrolled only 16,000 are practising, many because they cannot afford to do so! She needs also the home help in some form. Mr. Hayes Fisher, who had shown his sincere interest by travelling through the night from Liverpool to give the inaugural address, amused his audience by saying that though his Maternity Bill might be called a shabby little measure by some grown-ups, if the babies had votes they would poll for it—to a baby.

But we go on. Infant welfare and the housing question are inextricably intertwined. "If Christianity does not do away with the one-roomed tenement," Mrs. H. B. Irving said, quoting Mr. Moody, "the one-roomed tenement may do away with Christianity." In such places birth and death have sometimes to happen while the family life goes on. In the Central Hall Exhibition some rather lurid coloured prints showed homes as they should not be, while excellent drawings and models of town-planning showed what skill could do when directed by intelligent ideas of how life should be lived in the home. A large black coffin showed the effects of city life and housing—Birmingham 134 in 1,000 births, Bournemouth 44, and Letchworth 36. Miss Hayden put in a wise plea for the large number of good homes of artisans, often cleaner than those of more highly-placed people.

Not that these have no needs. All mothers need education—and continued education—in their craft. But they individually cannot alter circumstances which Society could change if the nation takes its peace campaign seriously. Zymotic and venereal diseases could be abolished, tuberculosis practically so, eventually. London fogs and the fly-plague could certainly be conquered. A Christian country could—and the magnificent work done in the American Army in France, as related by Lieut.-Colonel Adami, proves it—so uplift morals that human selfishness should not be paid for by the cruel sufferings of innocent babes. When it can be said that 1,200 abandoned infants have had to be refused by one institution alone there is surely something Pecksniffian in charging the Hun with being a baby-killer. There is more than one sense in which the wages of sin is death.

Perhaps of all the lines of advance the most immediate is the standardised and improved training of the health visitor, whom Mr. Hayes Fisher views as the foundation of present effort. He held in his hand some new instructions, to come into force when the Maternity Bill is law. We shall do well to take lessons from France. Her splendid efforts for the children during an exhausting war, as related by more than one speaker, shame us. Think of a mothercraft exhibition in Dunkirk, set up between two bombardments; of trade scholarships given to the promising child of a family that a new bread-winner may quickly be raised up; of the Mayor of Lens arranging for confinements in the cellars of hospitals while projectiles rain. Yet Dr. Raimondi asks, "During four years' invasion could we do more, do better?" and answers, "Yes, we can. *And we will.*"

