# Erratum

**Published:** 1918-07-20

**Authors:** 


### Erratum.

IN last week's article on Baby Week (p. 331), not *Bournemouth* but *Bournville* should have been given as having an infant mortality rate of 44.

